# Supplementary material for: Transcriptome-guided annotation and functional classification of long non-coding RNAs in Arabidopsis thaliana
Source: Sci Rep. 2022 Aug 18;12:14063. doi: 10.1038/s41598-022-18254-0 (PMC9388643; doi:10.1038/s41598-022-18254-0)
Supplement: Supplementary file 1 — Supplementary Information. [file 41598_2022_18254_MOESM1_ESM.pdf]

Supplementary information

Transcriptome-guided annotation and functional classification of long non-coding RNAs in *Arabidopsis thaliana*

Jose Antonio Corona-Gomez[1†], Evelia Lorena Coss-Navarrete[1†], Irving Jair Garcia-Lopez[1], Christopher Klapproth[2,3], Jaime Alejandro Pérez-Patiño[1] and Selene L. Fernandez-Valverde[1*]

[1] Unidad de Genómica Avanzada, Langebio, Cinvestav, 36824 Irapuato, Guanajuato, México

[2] Leipzig University, Bioinformatics Group, Department of Computer Science and Interdisciplinary Center of Bioinformatics, Härtelstraße 16-18, D-04107 Leipzig, Germany

[3] ScaDS.AI Leipzig (Center for Scalable Data Analytics and Artificial Intelligence), Humboldstrasse 25, D-04105, Leipzig, Germany

† Equal contributions

* Corresponding author - selene.fernandez@cinvestav.mx

Supplementary Datasets

Dataset S1 Name and position of all annotated lncRNAs in bed 12 format, as well as their mature length, CPAT score, genomic location, biotype and confidence level. (Private link: https://figshare.com/s/5d19c00590cbd7fe73a1)

Dataset S2 Redundant lncRNAs biotypes in bed12 format plus biotype in column number 13 (Private link: https://figshare.com/s/859d12a6da94ba62c9fa)

Dataset S3 Table of metadata and ID of all transcriptomes used in this project (Private link: https://figshare.com/s/70c23951cda438d0fe35)

Dataset S4 Table of *Tau* values for the tissue-specific lncRNAs (Private link: https://figshare.com/s/5c47f80a3b2929ac45e4)

Dataset S5 Table of the lncRNAs and genes that conform WGCNA modules (Private link: https://figshare.com/s/0ca9c981b318c96f5f7c)

Supplementary Tables

Table S1 Information of root and shoot *A. thaliana* transcriptomes generated

Sample Name	SRA Accession Number	% GC	Total reads	Remaining reads after filtering
At_shootR1_1	SRR16093081	46%	36075409	31423073
At_shootR1_2	SRR16093081	47%	36075409	31423073
At_shootR2_1	SRR16093080	46%	49835624	43879678
At_shootR2_2	SRR16093080	47%	49835624	43879678
At_rootR1_1	SRR16093079	45%	40506616	35421322
At_rootR1_2	SRR16093079	45%	40506616	35421322
At_rootR2_1	SRR16093078	44%	40231170	35455572
At_rootR2_2	SRR16093078	45%	40231170	35455572

Table S2 lncRNAs with known functions with high *Tau* values in a single tissue

Gene ID	Tissue	*Tau*	lncRNA
AT2G34655	petiole	0.657	*APOLO*
AT5G03545	root	0.583	*AT4*
AT5G03545	plant callus	0.572	*AT4*
AT4G06195	hypocotyl	0.81	*AT4G06195*
AT4G06195	cotyledon	0.788	*AT4G06195*
AT4G06195	whole plant	0.543	*AT4G06195*
AT4G08415	embryo	1	*AT4G08415*
AT5G01155	SAM	0.739	*AT5G01155*
AT5G01155	flower	0.699	*AT5G01155*
AT5G01155	silique	0.559	*AT5G01155*
AT5G01675	seed	0.902	*COOLAIR*
AT5G01675	embryo	0.707	*COOLAIR*
AT1G21529	plant callus	0.975	*DRIR*
AT4G16355	petal	0.946	*ELENA1*
AT1G08103	SAM	0.913	*FLINC*
AT2G35747	SAM	1	*HID1*
AT3G09922	plant callus	0.874	*IPS1*
AT3G09922	root	0.836	*IPS1*
AT3G09922	cotyledon	0.609	*IPS1*
AT5G00580	seedling	0.957	*MARS*
AT5G00580	root	0.868	*MARS*
AT2G27400	hypocotyl	0.596	*TAS1a*
AT2G27400	cotyledon	0.566	*TAS1a*
AT2G27400	SAM	0.542	*TAS1a*
AT2G27400	root	0.54	*TAS1a*

Supplementary Figures

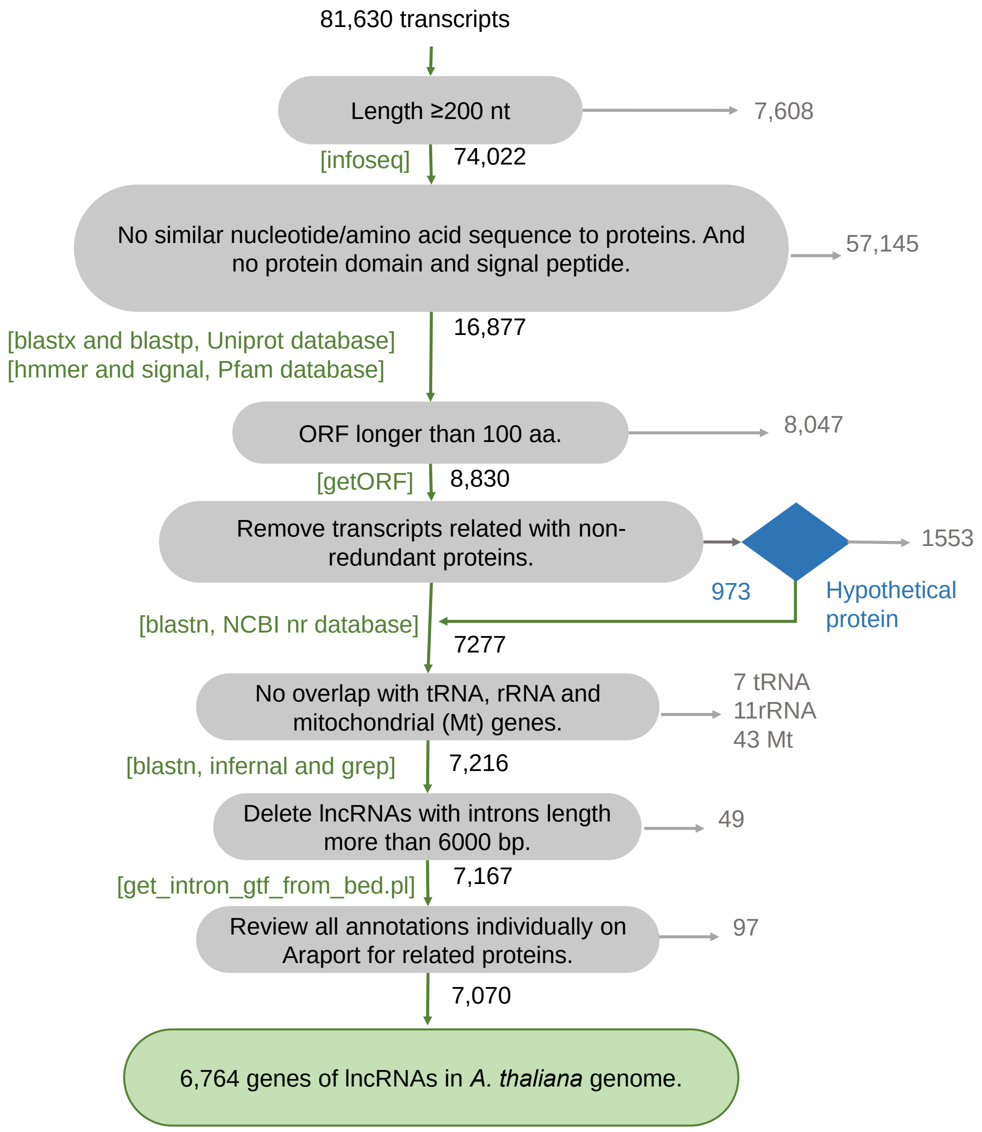

Figure S1. Diagram of filters used to annotate lncRNAs

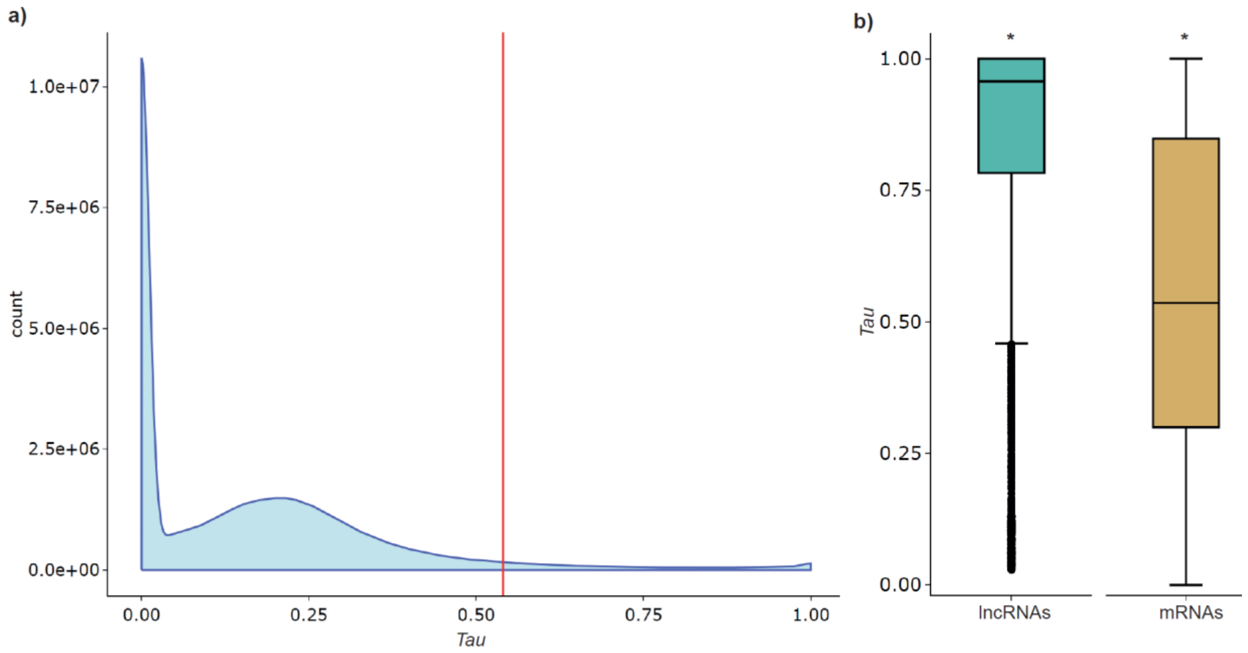

Figure S2. Distribution of values of *Tau.* a) Cut-off point of *Tau* (0.54) values. b) Boxplot with top values of *Tau* in lncRNAs and mRNAs. LncRNAs have significantly higher values than mRNAs (Wilcoxon text *p* = 2.2 e-16).

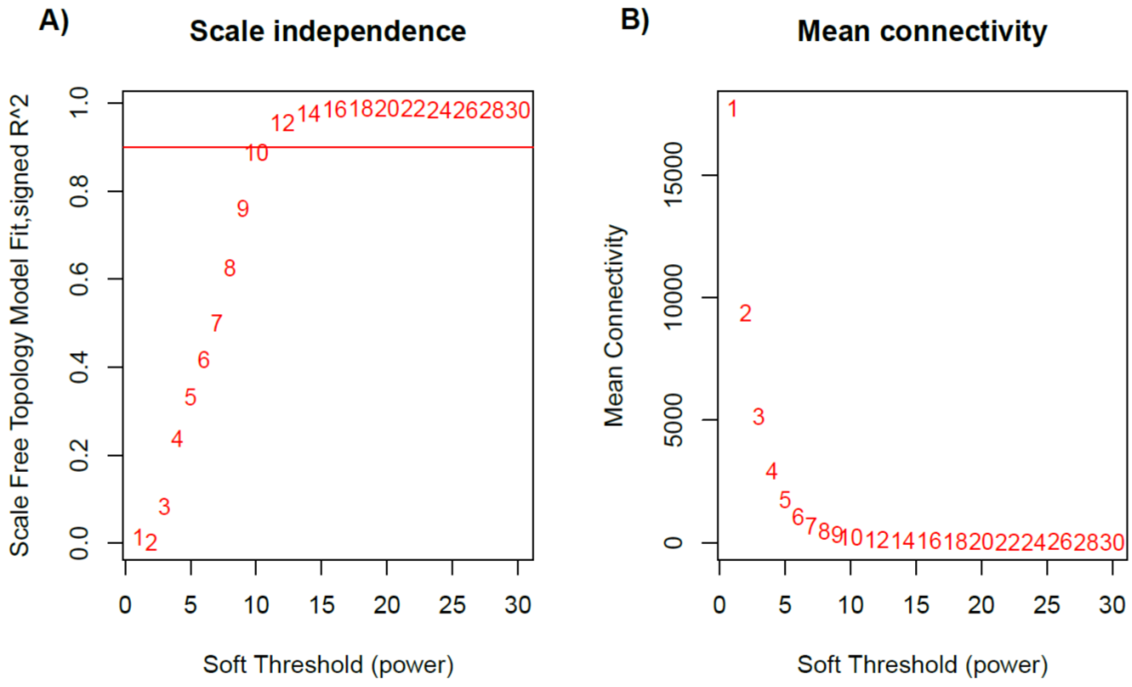

Figure S3. Network topology analysis. Various soft-thresholding powers (x-axis) are shown for the scale free topology fit index (A) and mean connectivity (B) (y axis). Based on these analyses we selected a power β soft threshold of 12. The red line in panel A corresponds to using an R^2 cut-off of h=0.9.

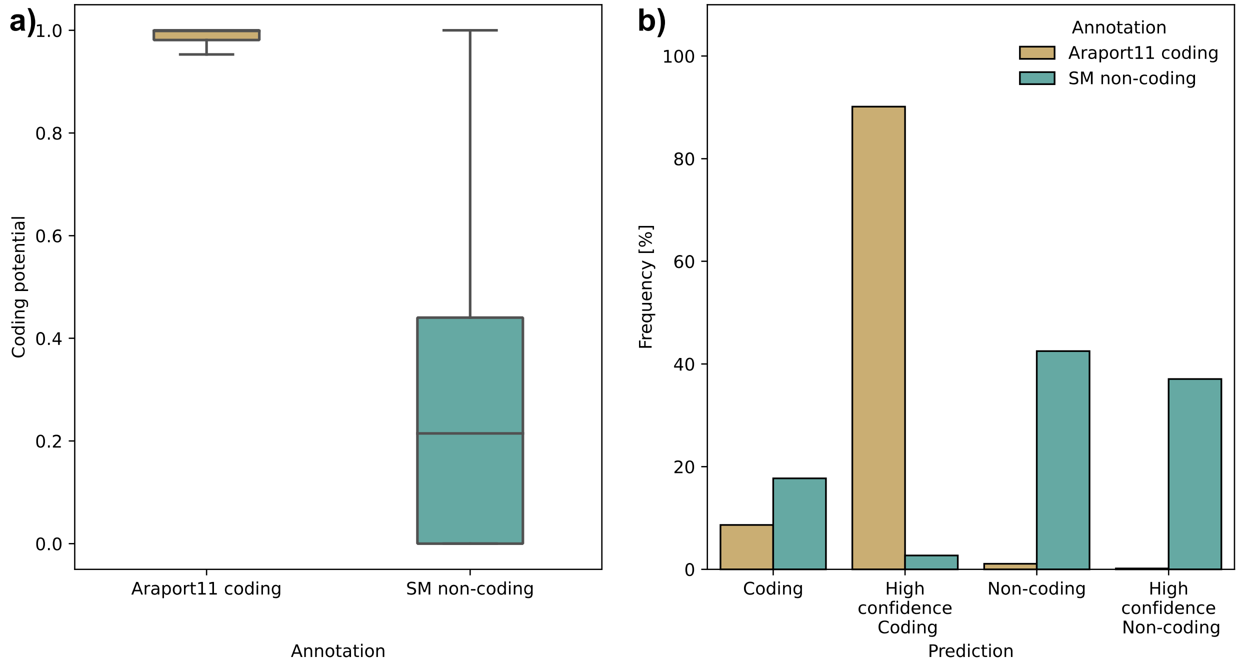

Figure S4. Coding potential assessment of Araport11 coding genes (ochre) and SM non-coding (aqua) a) Boxplot with CPAT coding potential values for both gene sets. LncRNAs have significantly lower coding potential values than mRNAs (Wilcoxon test p < 10 x e-15). b) Percentage of both classes classified by CPAT as coding (coding potential > 0.5), non-coding (coding potential <= 0.5), high confidence coding (coding potential >= 0.9) and high confidence non-coding (coding potential <= 0.1).

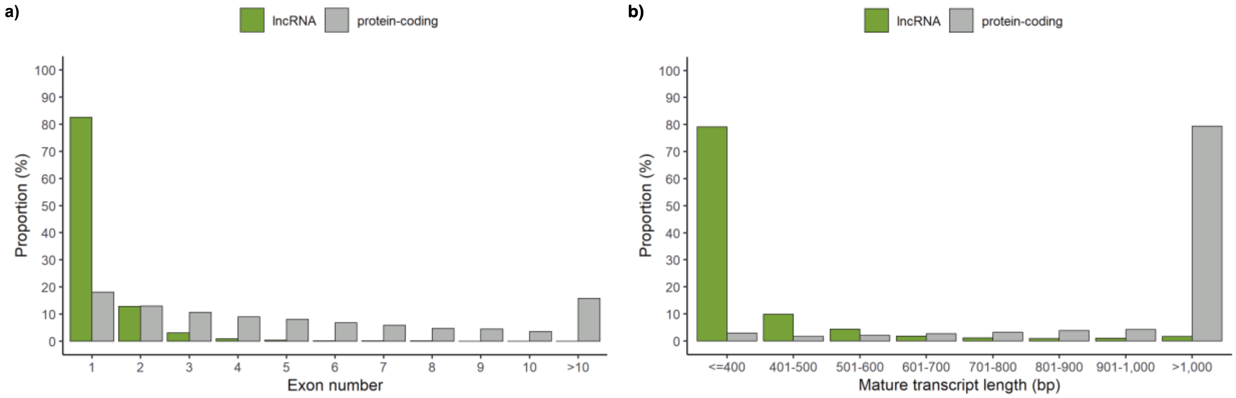

Figure S5. a) Proportion of the number of exons contained in the lncRNAs and coding genes. b) Proportion of the average size of the lncRNAs compared to the coding genes.

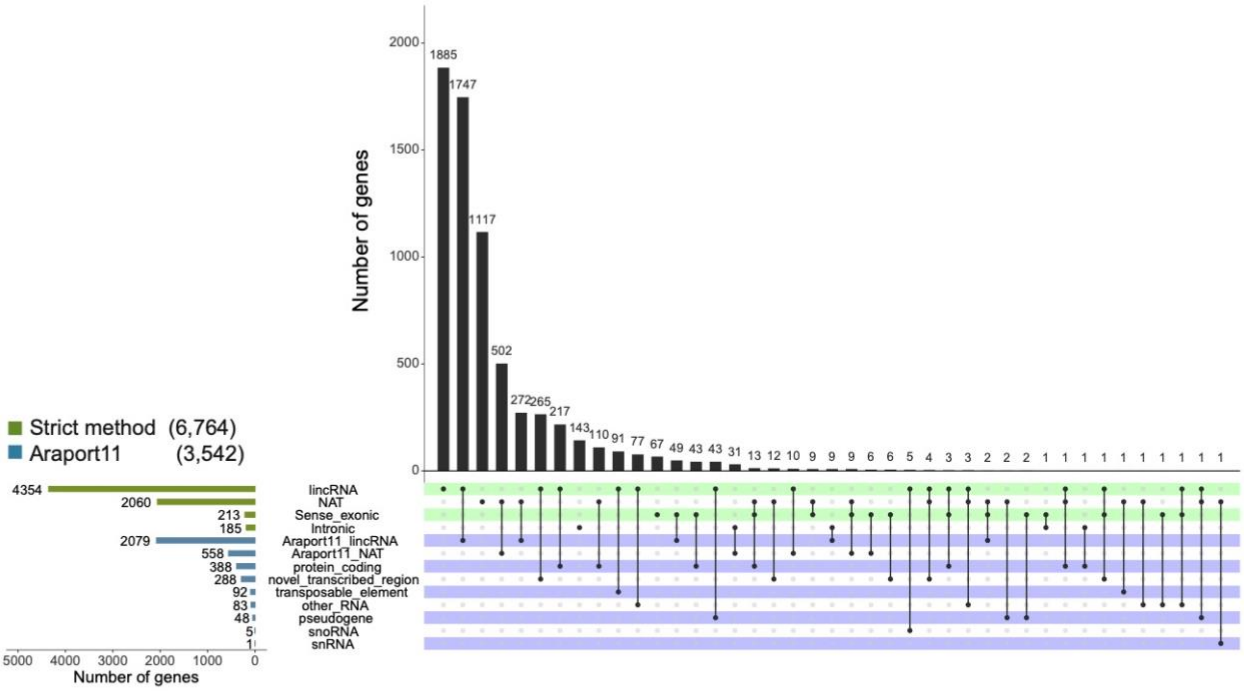

Figure S6. Comparison of each of the categories of lncRNAs present in Araport11 against the categories of the newly generated annotation.

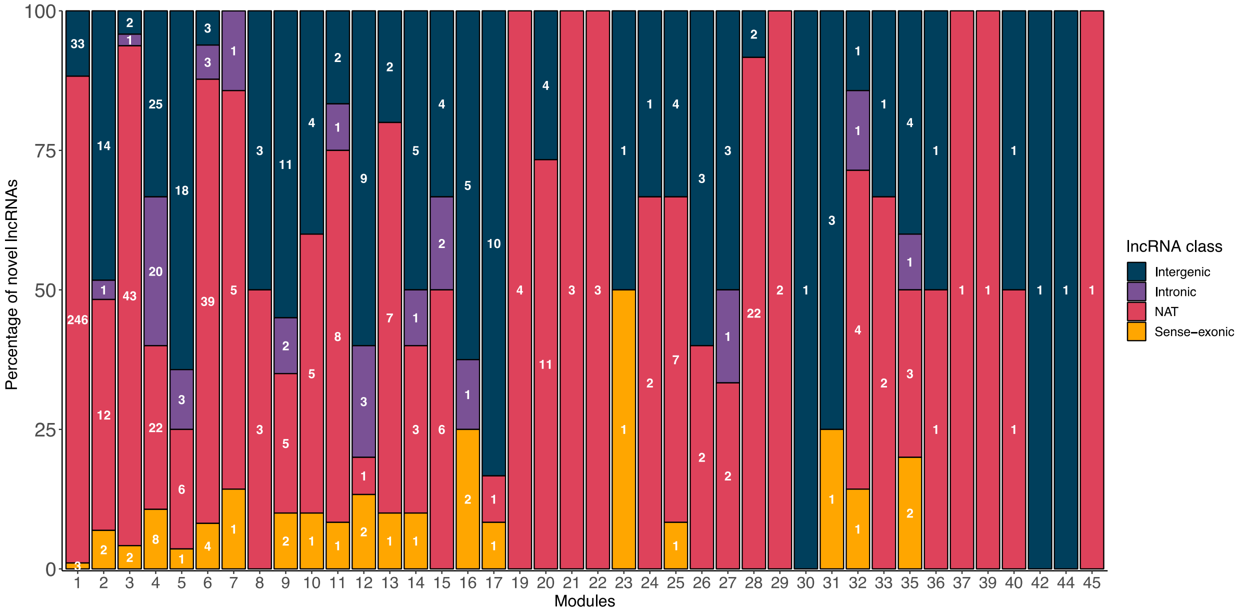

Figure S7. Novel lncRNAs per module. Proportion of novel lncRNAs (without annotation in Araport11) in each co-expression module and classified by biotype (Intergenic (lincRNA) - blue, Intronic - purple, NAT - pink, Sense-exonic - yellow).

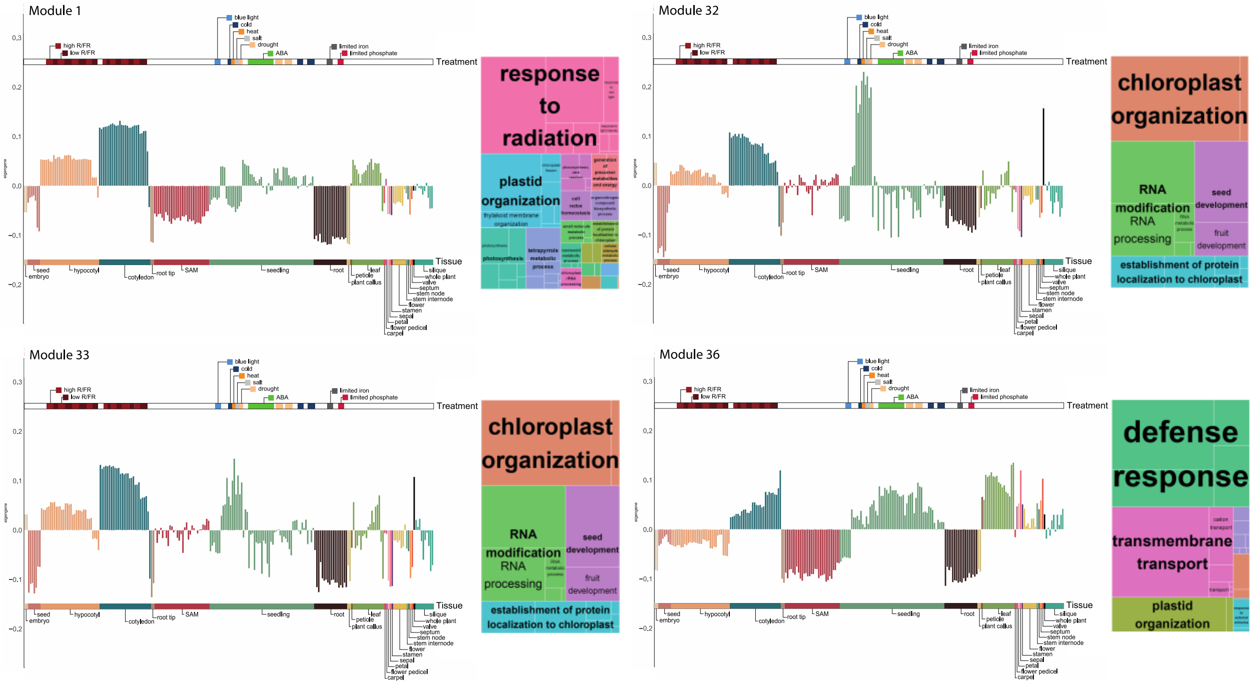

Figure S8. Eigengene expression per module for modules 1, 32, 33 and 36, chloroplast organization and photosynthesis functional category (4 modules with 409 lncRNAs).

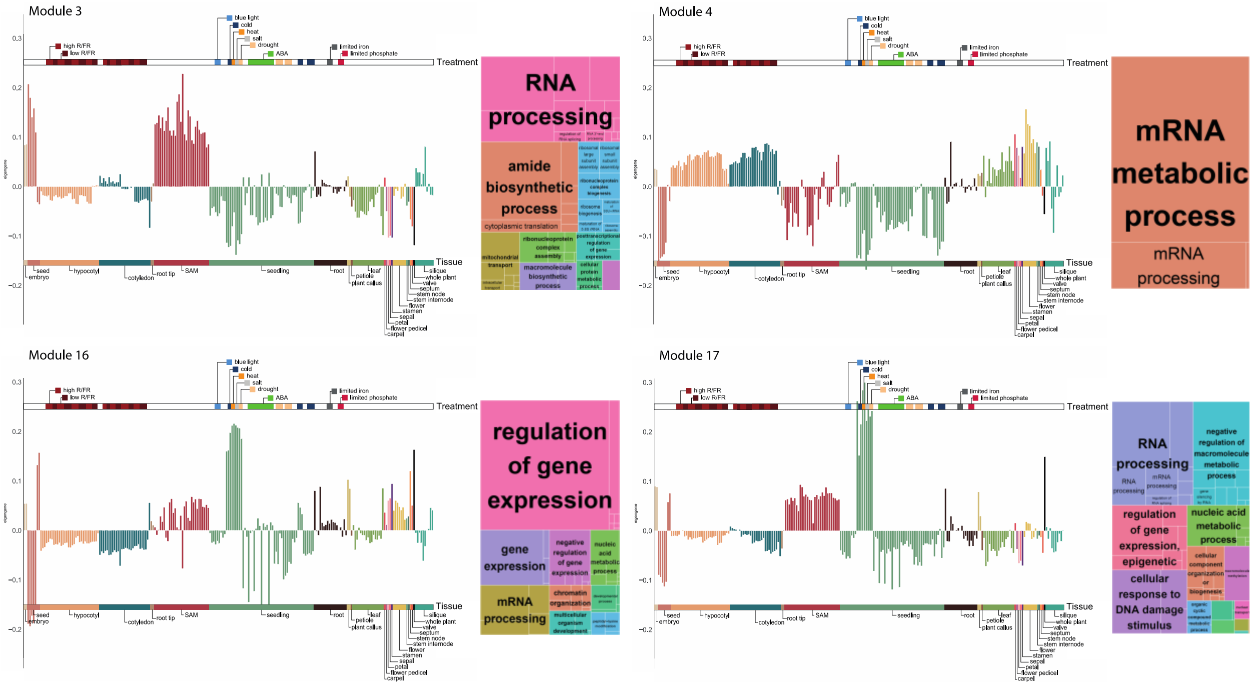

Figure S9. Eigengenes expression per module for modules 3, 4, 16 and 17; RNA regulation and transcription functional category (4 modules with 375 lncRNAs).

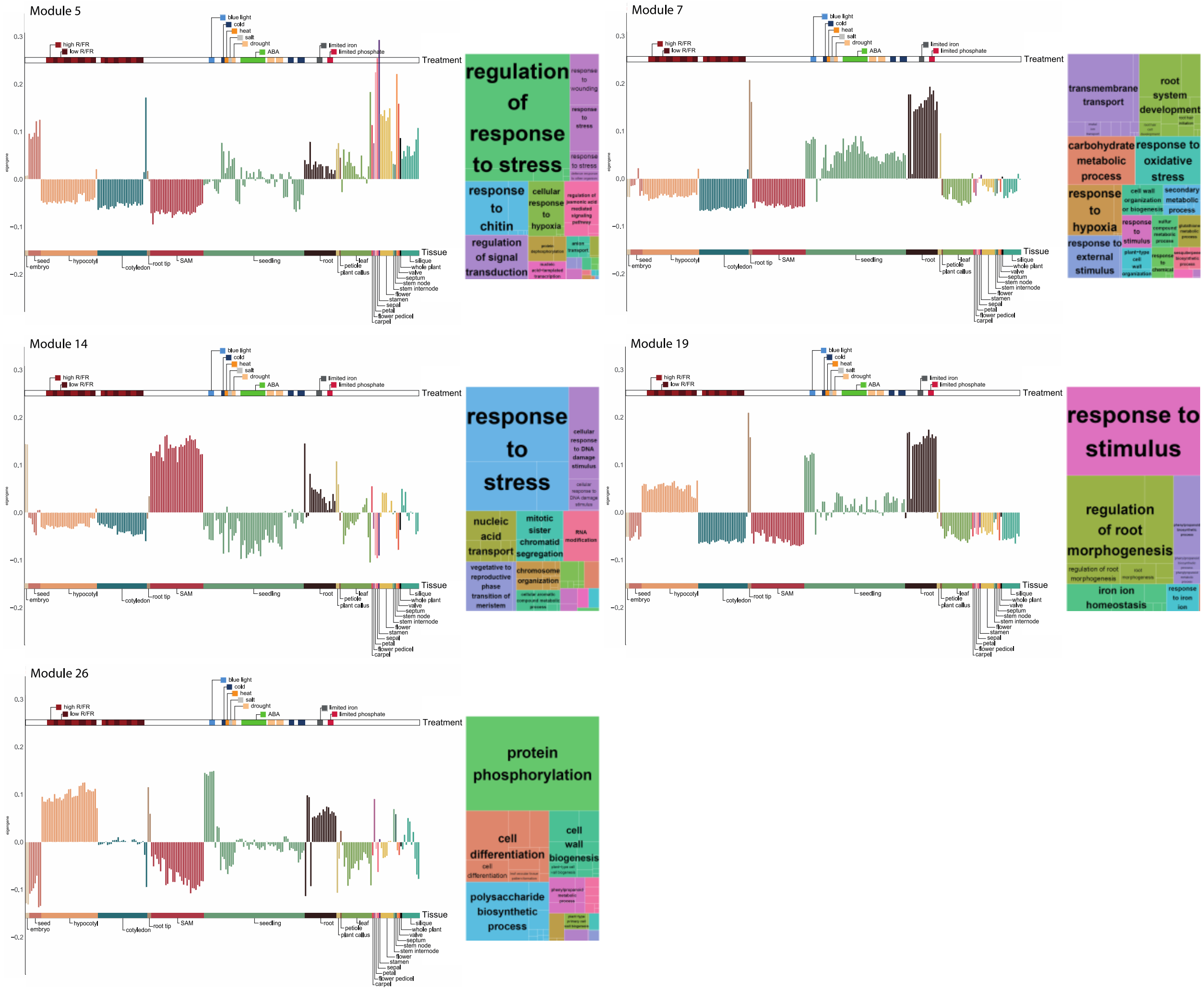

Figure S10. Eigengene expression per module for modules 5, 7, 14, 19 and 26; root development and response to root-related stress functional category (5 modules with 125 lncRNAs).

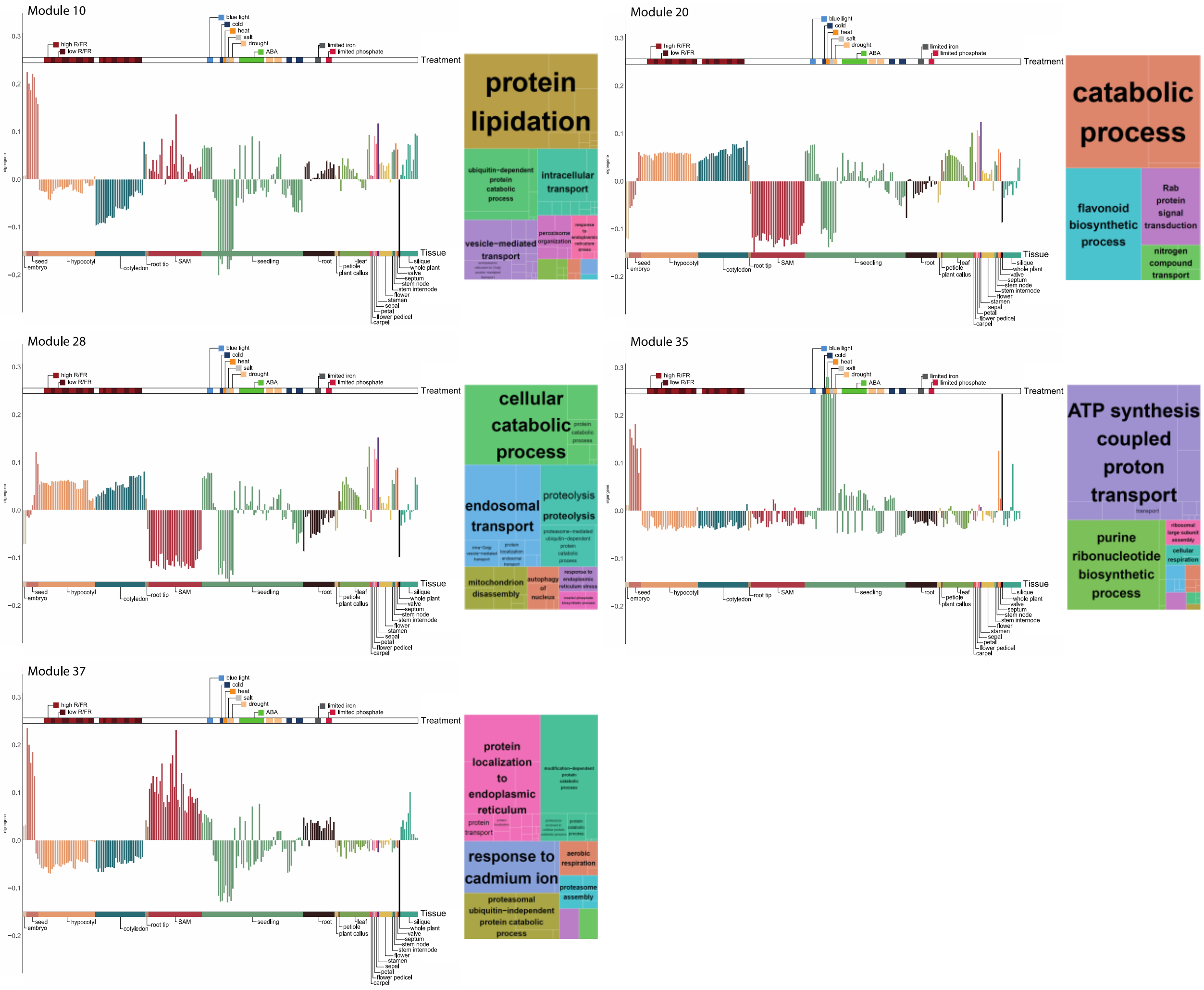

Figure S11. Eigengene expression per module for modules 10, 20, 28, 35 and 37; protein labeling and transport functional category (5 modules with 117 lncRNAs).

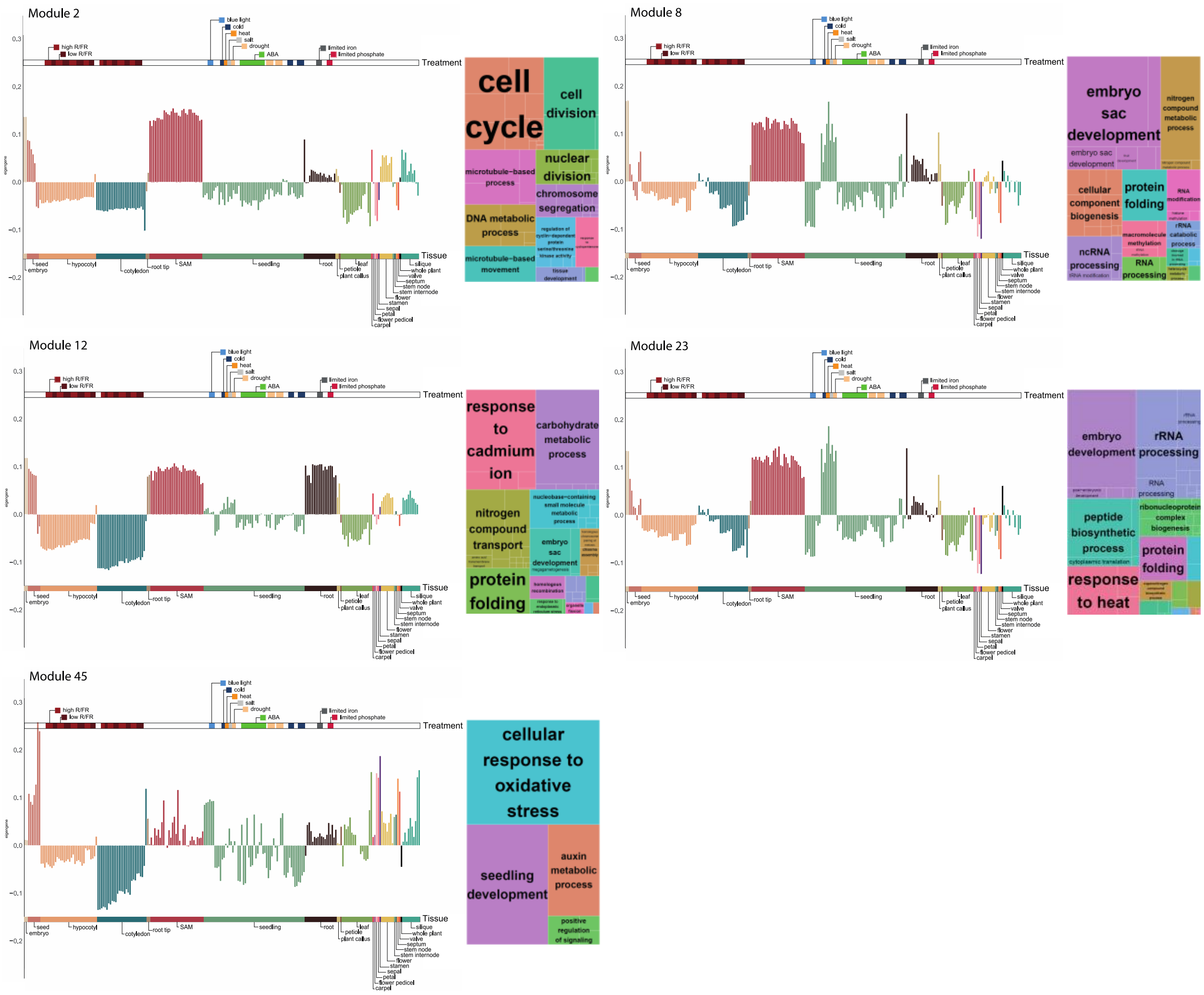

Figure S12. Eigengene expression per module for modules 2, 8, 12, 23 and 45; cell division functional category (5 modules with 112 lncRNAs).

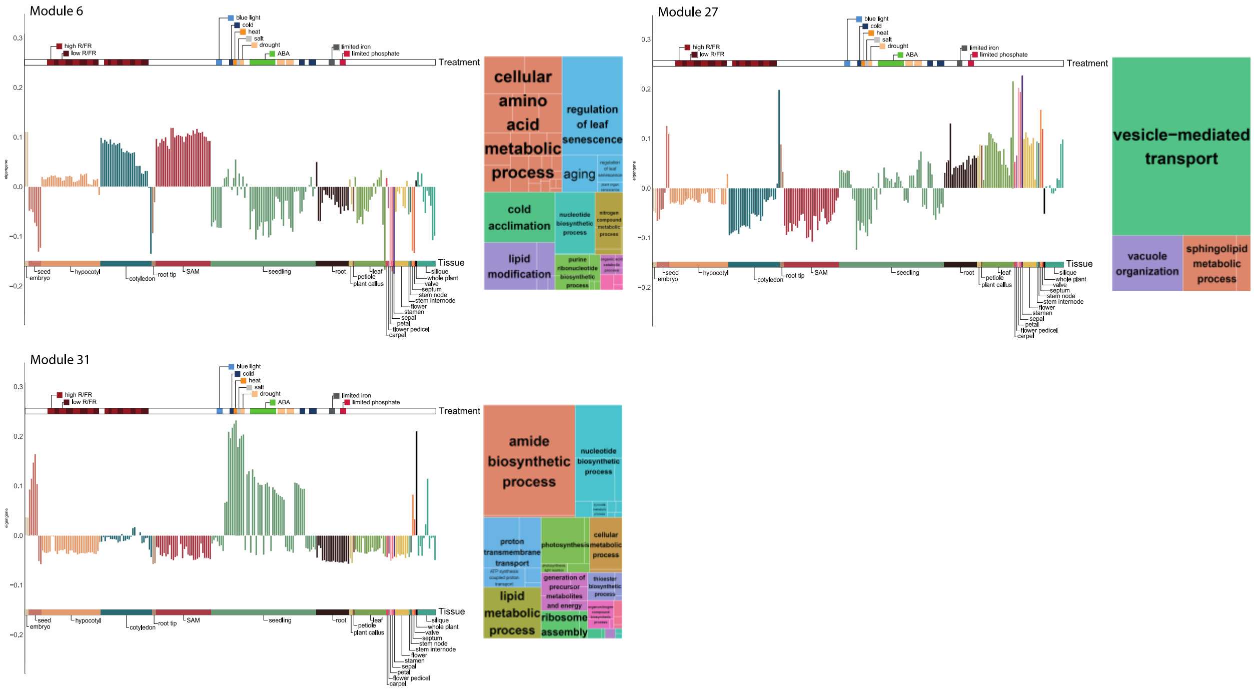

Figure S13. Eigengene expression per module for modules 6, 27 and 31; lipids and membranes functional category (3 modules with 97 lncRNAs).

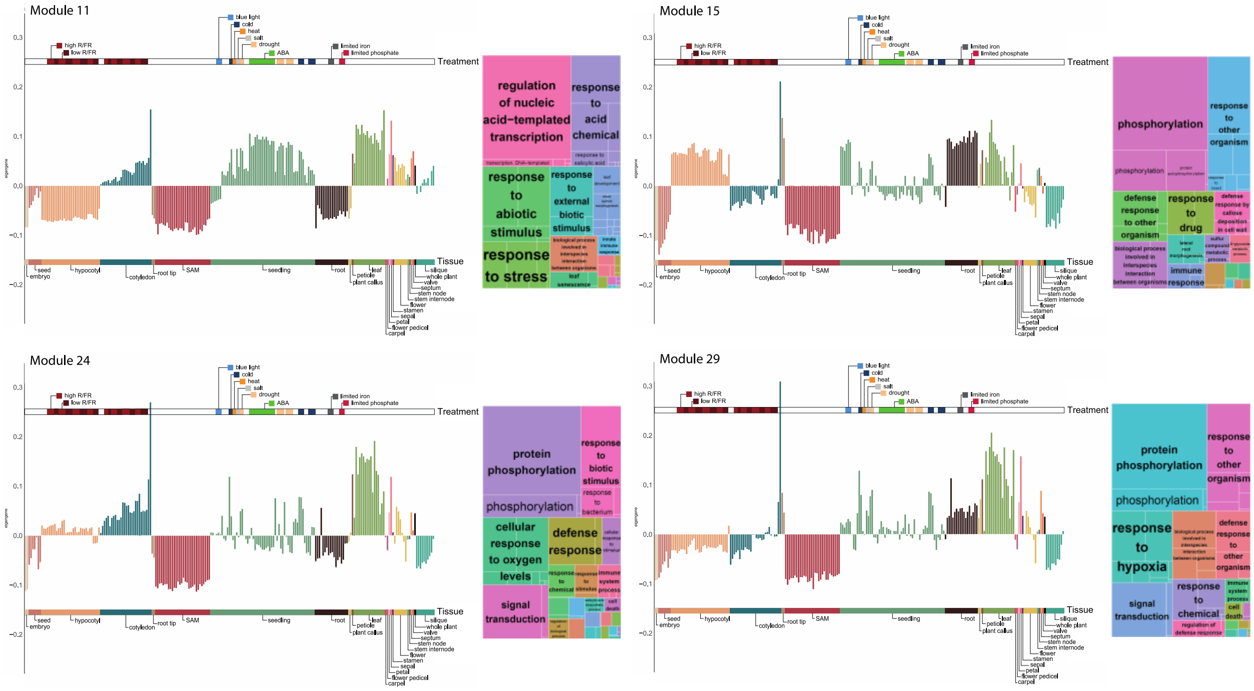

Figure S14. Eigengene expression per module for modules 11, 15, 24 and 29; response to pathogens functional category (4 modules with 72 lncRNAs).

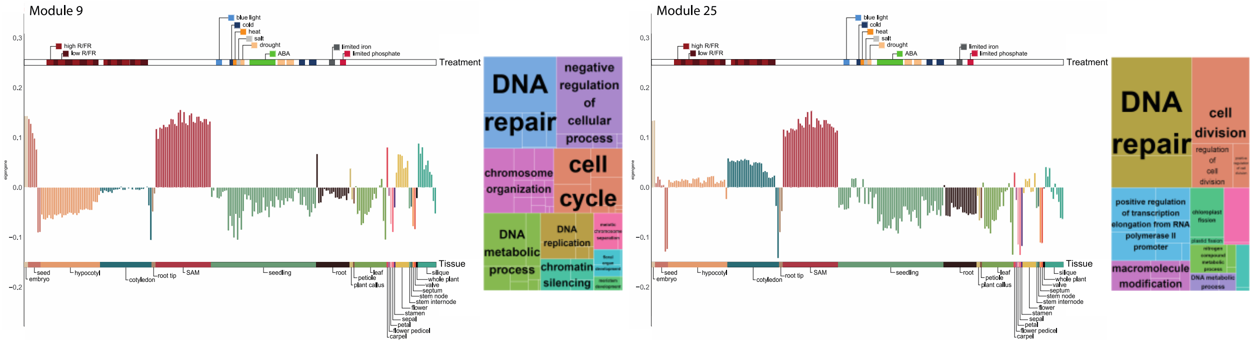

Figure S15. Eigengene expression per module for modules 9 and 25; DNA repair functional category (2 modules with 61 lncRNAs)

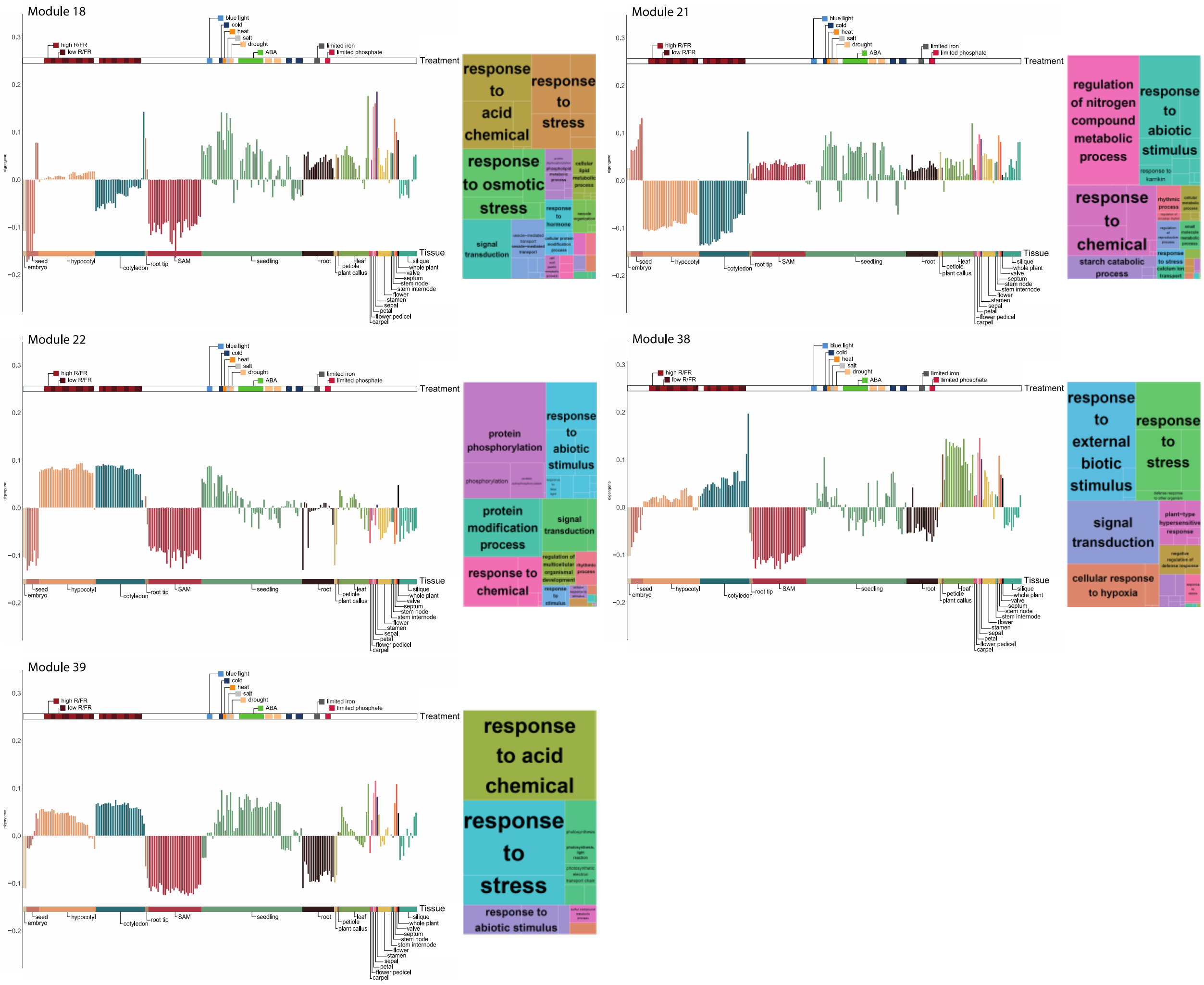

Figure S16. Eigengene expression per module for modules 18, 21, 22, 38 and 39; response to stress functional category (5 modules with 17 lncRNAs).
